# Cardiovascular risk factors among Ghanaian patients with HIV: A cross‐sectional study

**DOI:** 10.1002/clc.23273

**Published:** 2019-09-30

**Authors:** Lambert T. Appiah, Fred S. Sarfo, Mark D. Huffman, Samuel B. Nguah, Jonathan K. Stiles

**Affiliations:** ^1^ Komfo Anokye Teaching Hospital Kumasi Ghana; ^2^ Kwame Nkrumah University of Science & Technology School of Medicine and Dentistry Kumasi Ghana; ^3^ Northwestern University Feinberg School of Medicine Chicago Illinois; ^4^ The George Institute for Global Health Sydney Australia; ^5^ Morehouse School of Medicine Atlanta Georgia

**Keywords:** abdominal obesity, cardiovascular disease, CVD risk, diabetes mellitus, Ghana, high LDL‐C, highly active antiretroviral therapy, hypercholesterolemia, hypertension, hypertriglyceridemia, low HDL‐C, smoking

## Abstract

**Background:**

Cardiovascular disease (CVD) poses a significant cause of morbidity and mortality among people living with human immunodeficiency virus (HIV). However, data are limited on CVD risk burden among HIV patients in Ghana. We describe the age‐ and sex‐adjusted prevalence of CVD risk factors among HIV patients in Ghana.

**Methods:**

From January 2013 to May 2014, we identified eligible HIV patients 18 years and older, as well as uninfected adult blood donors presenting to the Komfo Anokye Teaching Hospital as controls. Using a standardized protocol, we collected demographic, clinical, laboratory, and electrocardiographic data. We created multivariable logistic regression models to compare the prevalence of abnormal risk factors between the two groups.

**Results:**

We recruited 345 patients with HIV (n = 173 on HAART, n = 172 not on HAART) and 161 uninfected adult blood donors. Patients with HIV were older (mean [SD] age: 41 [11] vs 32 [11] years) and were more likely to be female (72% vs 28%) than blood donors. Among patients on HAART, median (interquartile range) treatment duration was 17 (4‐52) months. The prevalence of hypertension, hypercholesterolemia, and diabetes mellitus among HIV patients was 9%, 29%, and 5%, respectively, compared with 5%, 15%, and 0.6% among uninfected blood donors. Smoking was the least prevalent CVD risk factor (1%‐2%). After adjustment for age, sex, and body mass index, HIV patients had a 10‐fold higher odds of prevalent diabetes compared with controls, (adjusted OR = 10.3 [95% CI: 1.2, 86.7]).

**Conclusion:**

CVD risk factors are common among HIV patients in Ghana, demonstrating the urgent need for creation and implementation of strategic CVD interventions.

## 1 INTRODUCTION

Human immunodeficiency virus/ acquired immune deficiency syndrome (HIV/AIDS) is the leading cause of death in Africa accounting for 20% of mortality. HIV prevalence is also high: although sub‐Saharan Africa (SSA) has only 11% of the world's population, 70% of all adults with HIV live in SSA.^1^ Antiretroviral therapy has substantially improved the prognosis for HIV‐infected patients^2^ with steadily declining HIV/AIDs‐related deaths within SSA following the dramatic scaling up of antiretroviral therapy and other priority HIV/AIDS interventions since 2004.^3^ Amidst falling HIV incidence rates, people living with HIV are living longer and are thus exposed to risks for comorbid conditions, especially cardiovascular diseases (CVD). HIV/AIDS, highly active antiretroviral therapy (HAART), and associated behaviors lead to 50% to 100% higher risk for CVD among individuals living with HIV compared with the general population.^4^, ^5^

The combination of HIV and CVD has created an epidemic that is projected to surpass infectious disease alone as the leading cause of mortality in SSA by 2030^6^ and presents a significant risk toward achieving the United Nations' Sustainable Development Goals 3.3 and 3.4 which aim to end the AIDS epidemic and to reduce the risk of premature deaths from noncommunicable diseases by one‐third by 2030, respectively.^7^

Routine assessment and monitoring of CVD risk among HIV patients globally is generally suboptimal^8^ with only one published report from Ghana, in particular.^9^ This current study sought to fill this gap in knowledge and to estimate the CVD burden of HIV patients presenting for routine visits at the largest HIV clinic in central Ghana. The ultimate aim is to drive policy and practice reforms in universal access to comprehensive, accessible, and quality health services to reduce HIV‐related morbidity and mortality.

## 2 METHODS

### 2.1 Study setting and recruitment

The study investigated HIV‐positive adults 18 years and older who received care at the Komfo Anokye Teaching Hospital (KATH) between January 2013 and May 2014. KATH is a 1200‐bed facility in Kumasi and the second‐largest teaching hospital in Ghana, serving approximately 4 million people in the Ashanti Region. KATH is affiliated with the School of Medicine and Dentistry (SMD) of the Kwame Nkrumah University of Science and Technology (KNUST). KATH's HIV clinic cares for approximately 6000 patients annually and is the largest HIV clinic within central and northern Ghana. The hospital offers general and specialized medical care and serves as a referral hospital for all regional and district hospitals in the Ashanti Region and to six out of 10 other regions across country. This study was approved by the Committee on Human Research, Publication and Ethics of the KNUST‐SMD.

We recruited consecutive adult HIV patients 18 years or older presenting to the HIV clinic for routinely scheduled visits and HIV‐negative adult blood donors presenting to the blood bank of the hospital to serve as controls. All participants provided written informed consent.

### 2.2 Study procedures

All participants completed a standardized questionnaire to capture data on demographics, socioeconomic position, medical history, and medication use by trained study personnel. At the time of interview, anthropometry, blood pressure, and 12‐lead electrocardiographic measurements were obtained. Weight and height were measured without shoes while wearing light clothes, and body mass index (BMI) was calculated as the weight in kilograms divided by the square of the height in meters. The abdominal circumference was measured as the narrowest circumference between the lower rib margin and anterior superior iliac crest above the umbilicus at exhalation. Participants had their systolic blood pressure (SBP) and diastolic blood pressure (DBP) measured after 5 minutes of rest in a seated position with their arms, back, and feet supported. The first and fifth Korotkoff sounds were registered to indicate SBP and DBP, respectively. Two blood pressure measures were obtained, and the mean was calculated. Hypertension was defined as SBP ≥ 140 mmHg, DBP ≥ 90 mmHg, or taking blood pressure lowering medications.

Blood samples were obtained from all participants through venipuncture. Serum concentrations of glucose, total cholesterol, HDL cholesterol, and triglycerides were determined using the enzymatic method in the Cobas Integra 400 (Roche). LDL cholesterol was calculated using the Friedewald formula. CD4 lymphocyte count was performed using Becton Dickinson FACSCalibur flow cytometer 4 color basic.

### 2.3 Study definitions

BMI of less than 18.5 kg/m^2^ defined underweight, 18.5 to 24.9 kg/m^2^ defined normal body weight, 25 to 29.9 kg/m^2^ defined overweight, and equal to or greater than 30 kg/m^2^ defined obesity. Abdominal obesity was defined as a waist circumference of ≥ 80 cm in females and ≥ 94 cm in males. Current or past smoking history was ascertained from the patient. Hypertension was defined as SBP ≥ 140 mmHg, DBP ≥ 90 mmHg, or self‐reported use of blood pressure lowering medication. Hypercholesterolemia was defined as total cholesterol ≥ 200 mg/dL (≥ 5.18 mmol/L) or self‐reported use of lipid lowering therapy. Hypertriglyceridemia was defined as triglycerides ≥ 150 mg/dL (≥ 1.7 mmol/L). Low HDL cholesterol was defined as HDL‐C ≤ 50 mg/dL (≤ 1.30 mmol/L) for women or ≤ 40 mg/dL (≤ 1.04 mmoL/L) for men. High LDL cholesterol was defined as LDL‐C ≥ 150 mg/dL (≥ 3.8 mmol/L). Diabetes mellitus (DM) was defined as a previous diagnosis of type 1 or 2 DM, at least two random blood glucose readings of ≥ 11.1 mmol/L, fasting plasma glucose reading of ≥ 7 mmol/L, or self‐reported use of a glucose‐lowering agent. Electrocardiogram (ECG) abnormalities were classified into major and minor forms using the Minnesota Coding system.

### 2.4 Statistical analysis

We first compared the demographics and clinical covariates of individuals with HIV infection and adult blood donors without HIV. Subsequently, we compared the prevalence of CVD risk factors for two groups before and after adjustment for demographics using multivariable logistic regression models that adjusted for age, sex, and BMI. We also explored differences among individuals with HIV who were and were not on baseline HAART. In all analyses, two‐sided *P*‐values of < .05 were considered statistically significant. We used Stata SE (version 13) for statistical analyses.

## 3 RESULTS

### 3.1 Characteristics of study population

**Figure 1:**
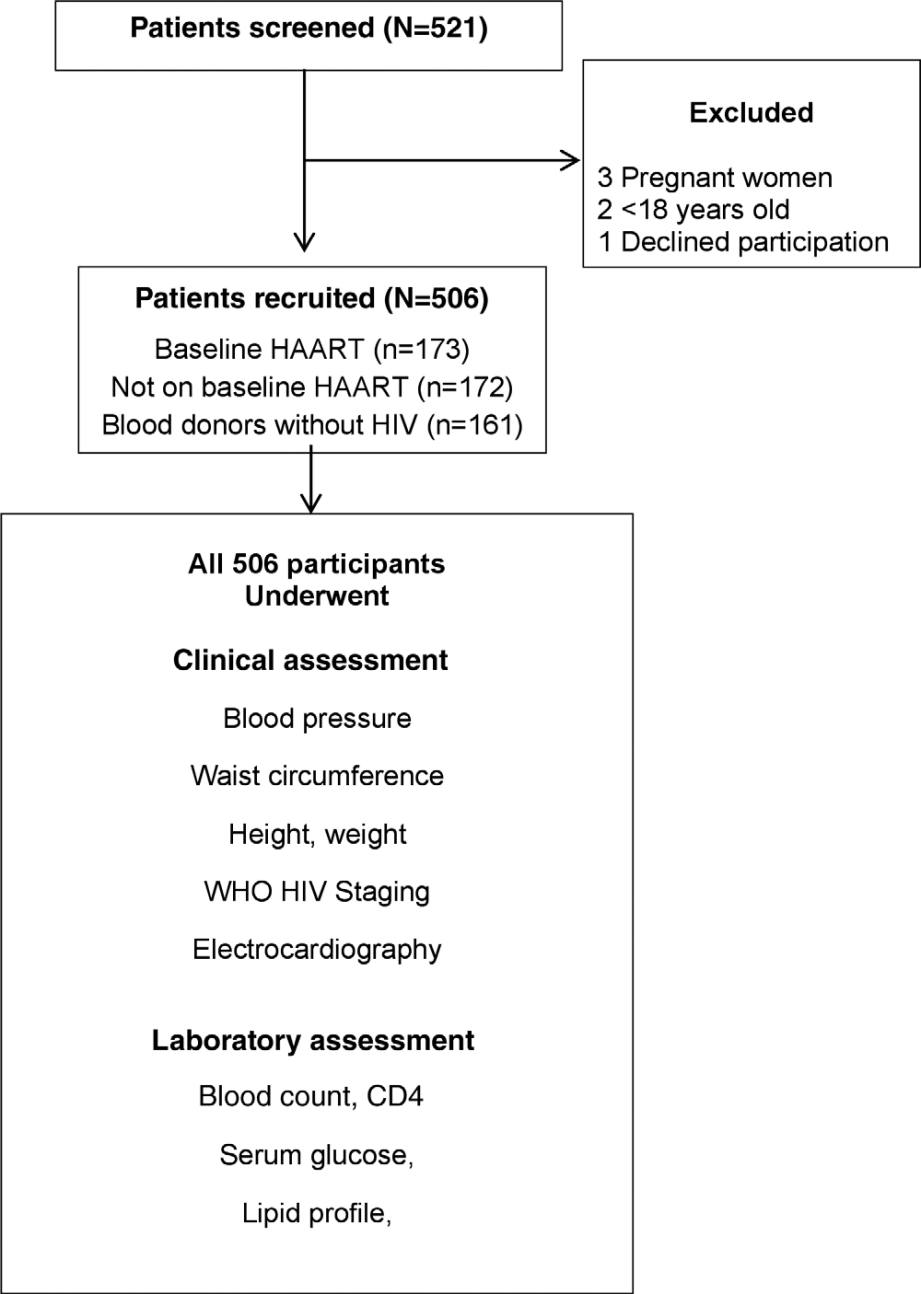
Flow chart of study participants

The flowchart of participants is shown in Figure 1, and baseline characteristics of the participants are outlined in Table 1. We recruited 345 patients with HIV and 161 adult blood donors without HIV. The sample included 172 (50%) patients on baseline HAART with median (interquartile range [IQR]) duration of treatment of 17 (4‐52) months, and 173 (50%) patients with HIV not on baseline HAART. Differences in baseline characteristics among patients with HIV on baseline HAART and not on baseline HAART are shown in Tables S1 and S2.

**Table 1:** Characteristics of study population among individuals with HIV and blood donors without HIV

Characteristic	Individuals with HIV (n = 345)	Blood donors without HIV (n = 161)	*P*‐value
Age, years, mean (SD)	41 ± 11	32 ± 10.5	< .001
Female, n (%)	249 (72)	45 (28)	< .001
Christianity, n (%)	312 (90)	141 (88)	.33
Employed, n (%)	292 (85)	126 (78)	.04
BMI, mean (SD) kg/m^2^	22 ± 4.8	26 ± 4.4	< .001
Hemoglobin, mean (SD) g/dL	10.7 ± 2	13 ± 1.4	.05
SBP, mean (SD) mmHg	112.6 ± 17.7	121.2 ± 12.3	< .001
DBP, mean (SD) mmHg	72.6 ± 13.2	75.8 ± 8.4	.002
Waist circumference, mean (SD) cm	74.1 ± 13.5	79.1 ± 11.9	< .001
Smoking, n (%)	2 (1.2)	6 (2.0)	.68
lipid profile			
Total cholesterol, mean (SD) mg/dL	169.6 ± 52.3	159.3 ± 37.6	.02
LDL‐c, mean (SD) mg/dL	93.3 ± 38.1	96.1 ± 30.8	.22
HDL‐c, mean (SD) mg/dL	45.1 ± 17.5	38.8 ± 11.3	< .001
Triglycerides mean (SD)	119.5 ± 58.5	111.3 ± 60.3	.09
Framingham risk score, n (%)			
Moderate to high predicted risk*, n (%)	28 (11)	8 (9)	.60
ECG abnormalities			
Major, n (%)	162 (47)	42 (26)	.001
Minor, n (%)	35 (10)	13 (8)	.54

*Note*: Major ECG abnormalities include left ventricular hypertrophy, right ventricular hypertrophy, atrial enlargement, bundle branch block, low voltages, ST‐T changes (eg, T wave inversions, ST elevation, or ST depressions). Minor ECG abnormalities include premature complexes, bradycardia, tachycardia, and axis deviation.

Abbreviations: BMI, body mass index; DBP, diastolic blood pressure; ECG, electrocardiography; HDL, high‐density lipoprotein; LDL, low‐density lipoprotein; SBP, systolic blood pressure.*This means FRS of 10 or more.

Patients with HIV were older (mean [SD] age = 41^11^ vs 32^11^ years, *P* < .001) and were more likely to be female (72% vs 28%, *P* < .001) than blood donors. Mean [SD] SBP (112.6 [17.7] mmHg vs 121.2 [12.3] mmHg, *P* < .001) and DBP (72.6 [13.2] mmHg vs 75.8 [8.4] mmHg, *P* = .002) were lower among HIV patients compared to the adult blood donors without HIV. Smoking was the least prevalent CVD risk factor with similar rates between groups (1%‐2%). Compared with patients with HIV not on baseline HAART, patients with HIV on HAART were more likely to be female (79% vs 66%, *P* = .008) and had a higher mean (SD) BMI (22.9 [5.4] kg/m^2^ vs 21.2 [4.1] kg/m^2^, *P* = .001), and a higher median (IQR) total absolute lymphocyte CD4 T cell counts (323 [120, 536] cells/μL vs 164 [56, 290] cells/μL). Major ECG abnormalities were more common among HIV patients compared to adult blood donors without HIV (47% vs 26%, *P* = .001). Most patients with HIV had the World Health Organization stage III disease, and over 98% of patients with HIV on baseline HAART used either a nevirapine‐ or efavarenz‐based regimen.

### 3.2 Comparison of CVD risk factors by HIV status

Table 2 shows the unadjusted and adjusted comparisons of CVD risk factors between patients with HIV and blood donors without HIV. The prevalence of hypertension, hypercholesterolemia, and DM among HIV patients was 9%, 29%, and 5%, respectively, compared with 5%, 15%, and 1% among HIV‐negative blood donors. After adjustment for age, sex, and BMI, individuals with HIV had a 10‐fold higher odds of prevalent diabetes compared with controls (adjusted OR = 10.3 [95% CI: 1.2, 86.7]). Low HDL‐C prevalence among HIV patients was 53% compared with 67% among HIV‐negative blood donors (adjusted OR = 0.4 [95% CI: 0.2, 0.8]). The prevalence of more than one abnormal risk factor was 56% among HIV patients compared to 38% among HIV‐negative blood donors (adjusted OR = 1.5 [95% CI: 0.9, 2.6]).

**Table 2:** Cardiovascular risk factors among individuals with HIV and blood donors without HIV

		Unadjusted				
	Individuals with HIV	Blood donors without HIV	Adjusted			
CVD risk factor	N = 345	N = 161	OR (95%CI)	*P*‐value	OR (95%CI)	*P*‐value
Hypertension, n (%)	30 (8.7)	8 (5.0)	1.8 (0.8‐4.1)	.14	1.0 (0.4‐2.7)	.97
Hypercholesterolemia, n (%)	83 (29.0)	24 (15.4)	2.2 (1.3‐3.6)	.002	1.6 (0.8‐3.0)	.18
Diabetes mellitus, n (%)	15 (5.0)	1 (0.6)	7.9 (1.0‐60.1)	.05	10.3 (1.2‐86.7)	.03
Hypertriglyceridemia, n (%)	70 (26.0)	31 (21.1)	1.3 (0.8‐2.1)	.26	1.2 (0.6‐2.4)	.52
High LDL‐C, n (%)	20 (7.6)	5 (3.4)	2.3 (0.9‐6.4)	.10	1.9 (0.6‐6.3)	.29
Low HDL‐C, n (%)	148 (53.2)	99 (66.9)	0.5 (0.4‐0.9)	.007	0.4 (0.2‐0.8)	.01
Smoking, n (%)	6 (2.0)	2 (1.2)	1.4 (0.3‐7.0)	.68	3.2 (0.5‐20.8)	.23
Abdominal obesity, n (%)	67 (19.4)	31 (19.2)	1.0 (0.6‐1.6)	.97	0.5 (0.2‐1.1)	.09
Any abnormal risk factor	296 (85.8)	128 (79.5)	1.6 (1.0‐2.5)	.08	1.0 (0.5‐1.9)	.96
> 1 CVD risks	194 (56.2)	62 (38.5)	2.0 (1.4‐3.0)	< .001	1.5 (0.9‐2.6)	.1
> 3 CVD risks	15 (4.3)	3 (2)	2.4 (0.7‐8.4)	.17	2.6 (0.6‐11)	.17

*Note*: Adjusted parameters are adjusted for age, sex, and body mass index. Hypercholesterolemia was defined as total cholesterol ≥ 200 mg/dL (≥ 5.18 mmol/L) or self‐reported use of lipid lowering therapy. Hypertriglyceridemia was defined as triglycerides ≥ 150 mg/dL (≥ 1.7 mmol/L). Low HDL cholesterol was defined as HDL‐C ≤ 50 mg/dL (≤ 1.30 mmol/L) for women or ≤ 40 mg/dL (≤ 1.04 mmoL/L) for men. High LDL cholesterol was defined as LDL‐C ≥ 150 mg/dL (≥ 3.8 mmol/L). Abdominal obesity was defined as a waist circumference of > 80 cm in females and > 94 cm in males.

Abbreviations: CVD, cardiovascular disease; HDL‐C, high‐density lipoprotein cholesterol; LDL‐C, low‐density lipoprotein cholesterol; OR, odds ratio.

### 3.3 Comparison of CVD risk factors by HIV treatment status

Table 3 presents the unadjusted and adjusted comparisons of CVD risk factors between individuals with HIV on baseline HAART and individuals with HIV not on baseline HAART. Compared with patients with HIV on baseline HAART, patients with HIV not on HAART had a lower odds of hypercholesterolemia (17% vs 39%, adjusted OR = 0.4 [95% CI: 0.2, 0.7]) and abdominal obesity (11% vs 28%, adjusted OR = 0.4 [95% CI: 0.4, 0.8]). Three out of every five (61%) HIV patients on HAART had two or more abnormal CVD risk factors compared with one out of every two (52%) HIV patients not on HAART (adjusted OR = 0.8 [95% CI: 0.5, 1.3]). Compared to HIV‐negative blood donors, HIV patients with CD4 count greater or equal to 350 had a 2.3 and 4.5 higher odds of having more than one and more than three CVD risks, respectively (Table S4).

**Table 3:** Cardiovascular risk factors and baseline HAART among individuals with HIV

	HIV treatment status		Unadjusted		Adjusted	
CVD risk factor	HAART (n = 173)	Not on HAART (n = 172)	OR (95% CI)	*P*‐value	OR (95% CI)	*P*‐value
Hypertension, n (%)	13 (7.5)	17 (10.0)	1.3 (0.6‐2.9)	.44	2.2 (1.0‐5.0)	.06
Diabetes mellitus, n (%)	7 (4.6)	8 (4.9)	1.1 (0.4‐3.0)	.89	1.2 (0.4‐4.0)	.76
Hypercholesterolemia, n (%)	59 (38.6)	24 (17.4)	0.3 (0.2‐0.6)	< .001	0.4 (0.2‐0.7)	.002
Hypertriglyceridemia, n (%)	37 (26.6)	33 (25.4)	0.9 (0.5‐1.6)	.82	1.0 (0.6‐1.8)	.97
High LDL‐C, n (%)	13 (9.5)	7 (5.5)	0.6 (0.2‐1.4)	.22	0.5 (0.2‐1.5)	.24
Low HDL‐C, n (%)	61 (42.7)	87 (64.4)	2.4 (1.5‐4.0)	< .001	2.3 (1.4‐3.8)	.001
Smoking, n (%)	3 (1.7)	3 (1.7)	1.0 (0.2‐5.0)	.99	0.8 (0.2‐4.5)	.83
Abdominal obesity, n (%)	49 (28.3)	18 (10.5)	0.3 (0.2‐0.5)	< .001	0.4 (0.2‐0.8)	.01
Any abnormal risk factor	149 (86.1)	147 (85.5)	0.9 (0.5‐1.7)	.86	1.0 (0.6‐2.0)	.90
> 1 CVD risks	105 (60.7)	89 (51.7)	0.7 (0.5‐1.0)	.09	0.8 (0.5‐1.3)	.34
> 3 CVD risks	8 (4.7)	7 (4.0)	0.9 (0.3‐2.5)	.80	1.5 (0.5‐5.0)	.46

*Note*: Adjusted parameters are adjusted for age, sex, and body mass index. Hypercholesterolemia was defined as total cholesterol ≥ 200 mg/dL (≥ 5.18 mmol/L) or self‐reported use of lipid lowering therapy. Hypertriglyceridemia was defined as triglycerides ≥ 150 mg/dL (≥ 1.7 mmol/L). Low HDL cholesterol was defined as HDL‐C ≤ 50 mg/dL (≤ 1.30 mmol/L) for women or ≤ 40 mg/dL (≤ 1.04 mmoL/L) for men. High LDL cholesterol was defined as LDL‐C ≥ 150 mg/dL (≥ 3.8 mmol/L). Abdominal obesity was defined as a waist circumference of > 80 cm in females and > 94 cm in males.

Abbreviations: CVD, cardiovascular disease; HDL‐C, high‐density lipoprotein cholesterol; LDL‐C, low‐density lipoprotein cholesterol; OR, odds ratio.

## 4 DISCUSSION

### 4.1 Summary of results

We assessed the burden of CVD risk factors among HIV patients routinely scheduled for clinic visits compared to adult blood donors who were HIV negative at the second largest teaching hospital in Ghana and found that HIV patients with CD4 count above 350 had a 2.3 times higher odds of having more than one CVD risk factors compared with adult blood donors without HIV. Again, HIV infection was significantly associated with higher odds of diabetes prevalence relative to blood donors. Similarly when compared to HIV patients not on HAART, we observed that HIV patients on HAART tendered to have a higher total cholesterol, higher HDL‐C levels, and a higher BMI possibly because their HIV was better managed, which led to less wasting and HIV‐related catabolism.

### 4.2 Comparison with previous research

DM was prevalent in 5% of patients with HIV in this study, which is similar to rates ranging from 4% to 8% reported in seven Latin American countries and South Africa, respectively.^10^, ^11^ The prevalence of diabetes in the present study was similar to reported rates in the general Ghanaian population,^12^, ^13^ but lower than rates among individuals with HIV in Nigeria (11% prevalence) and Senegal (15% prevalence), respectively.^14^, ^15^ In these studies, patients had been on HAART for a longer duration and on predominantly protease inhibitor‐based regimes, which was very low in the current study (1%). Protease inhibitors increase the risk for dysmetabolic states, which contributes to the increased incidence of diabetes in the HIV population.^16^, ^17^ As the use and availability of protease inhibitors becomes more widespread in Ghana and other low‐ and middle‐income countries, the burden of diabetes among individuals on HAART could substantially increase in the near future. The low prevalence of DM (1%) observed among blood donors without HIV could likely be due, at least in part, to the recruitment of volunteers who were younger and healthier than patients with HIV. On the other hand, this observation could further support the growing body of evidence suggesting that although obesity plays a role, HIV‐specific factors may allow diabetes to develop at a lower threshold of adiposity than in individuals without HIV, driven in part via the cycle of chronic inflammation, lipodystrophy, and its associated adipokine imbalance.^18^, ^19^, ^20^ Again HIV infection may be associated with resistance to fibroblast growth factors 12 and 19 which help maintain glucose homeostasis in adipose tissue.^21^ Future studies therefore needed to fully elucidate the pathophysiology of these HIV‐specific biomarkers that drive early development of diabetes among HIV patients.

There was no difference in the prevalence of hypercholesterolemia between patients with HIV and blood donors without HIV after adjustment. However, hypercholesterolemia was more common among patients with HIV on baseline HAART compared with patients with HIV not on baseline HAART, as has been demonstrated in Nigeria and Ethiopia.^22^, ^23^, ^24^ Nevirapine‐ and efavarenz‐based regimens (which are nonnucleoside reverse transcriptase inhibitors [NNRTIs]) have been demonstrated to increase the risk for hypercholesterolemia^25^ and could partially explain why hypercholesterolemia was more common among HIV patients on baseline HAART in this study. Nevirapine‐ and efavarenz‐based HAART regimens can induce lipolysis of subcutaneous fat while inhibiting lipogenesis and adipocyte differentiation resulting in mitochondrial toxicity and perturbations in lipid metabolism.^26^ Treatment of HIV itself likely reduces catabolism that lowers cholesterol levels among individuals not previously on HAART. Other potential contributory factors to the observed hypercholesterolemia among HIV patients on HAART include dietary or behavioral and genetic factors, which were not assessed in this study. The NNRTIs such as efavarenz also tend to have more favorable lipid profiles such as increased levels of HDL‐C,^27^ which can account for the significantly lower proportion of low‐HDL levels seen among HIV patients compared to blood donors without HIV.

Abdominal obesity was commonly seen among patients with HIV on baseline HAART compared with patients with HIV not on baseline HAART, even after adjusting for confounders; a similar trend was also observed for BMI. Plausible reasons include the improved immune system function with initiating HAART, deliberate attempts by patients to gain weight to obscure their HIV diagnosis, and the syndrome of fat redistribution that is associated with the use of HAART including zidovudine.^28^ These results may also mirror the rising trend in weight gain in the general Ghanaian population.^29^

Hypertriglyceridemia and high LDL‐C levels were similar across the various study groups, with similar results reported within other studies in SSA.^30^, ^31^, ^32^ The relatively low hypertriglyceridemia prevalence (26% among patients with HIV), even in the presence of relatively high prevalence of low HDL‐C (53% among patients with HIV), suggests the triglyceride paradox phenomenon, which has been observed in black populations and people of African descent. This paradox results from increased lipoprotein lipase (LPL) activity, low levels of apolipoprotein CIII activity, and a LPL activity that is uninhibited by insulin resistance in blacks compared to Caucasians, culminating in a greater clearance of triglyceride‐rich lipoproteins in blacks than whites.^33^, ^34^, ^35^, ^36^

The prevalence of hypertension in this study was 9% among patients with HIV infection. Comparison with previous data showed a similar reported prevalence of between 8% and 12% among Nigerians with similar patient characteristics.^14^ A larger study of > 12 000 HIV patients in Kenya with similar baseline characteristics (M:F ratio of 1:2.5) showed a similar hypertension prevalence of 11% for men and 7% for women.^37^ A higher prevalence has been reported, however, in both a Senegalese cohort (28%) and a recent Ghanaian cohort (40%), which may be due to differences in baseline age and sex.^9^, ^15^, ^38^ These findings underscore the fact that the burden of hypertension is likely to rise with an aging HIV population and should attract proportionate public health and clinical response to curb the projected increase in prevalence and its associated disease burden.

A smoking prevalence of 2% among patients with HIV and 1% prevalence in blood donors without HIV likely reflect the low smoking habits of Ghanaians, in general when compared to its West African counterparts.^39^

### 4.3 Strengths and limitations

This study is one of the first few studies describing the prevalence of abnormal undiagnosed CVD risk factors within the Ghanaian HIV population and will help contribute to accruing data and ongoing debate on the burden of CVD and the need to integrate CVD care in the routine care of HIV patients. On the other hand, this study also has inherent limitations, including a nonrepresentative sampling frame, its cross‐sectional study design, and baseline differences between patients with HIV and adult blood donors without HIV that raise the possibility of selection bias. Our data were collected between 2013 and 2014, and both CVD risk and HAART regimens may have changed over time. Again, there was no information on HIV viremia in this study and we also acknowledge that HAART may not perfectly stratify viral suppression possibly accounting for differences in dyslipidemia prevalence between groups. Nevertheless, this study remains relevant by providing baseline data for future research on CVD risk among individuals with HIV in Ghana.

## 5 CONCLUSION

This study demonstrates that abnormal undiagnosed CVD risk factors including obesity, hypertension, hypercholesterolemia, and DM were common and that HIV patients with CD4 count above 350 had higher odds of having more than one CVD risk factors compared to adult blood donors without HIV. HIV physicians and systems must scale up efforts to consolidate gains made so far with HAART by routinely assessing CVD risk factors for better prevention, treatment, and control.

## CONFLICT OF INTEREST

The authors have no conflict of interest to declare.

## Supporting information


**Appendix S1** Supporting information

**Table S1** Distribution of lipids, obesity, and blood pressure by treatment status

**Table S2** Basic characteristics of study population

**Table S3** Cardiovascular risk factors among HIV individuals on HAART with CD4 count > 350 and blood donors without HIV

**Table S4** Cardiovascular risk factors of HIV individuals with CD4 count < 350 and HIV individuals with CD4 count >= 350 compared to blood donors without HIV
