# Supplementary material for: Cardiovascular risk factors among Ghanaian patients with HIV: A cross‐sectional study
Source: Clin Cardiol. 2019 Sep 30;42(12):1195–201. doi: 10.1002/clc.23273 (PMC6906980; doi:10.1002/clc.23273)
Supplement: Supplementary file 1 — Appendix S1 Supporting information [file CLC-42-1195-s001.docx]

**Supplementary Sheet**

| **SSTable 1:Distribution of lipids, obesity and blood pressure by treatment status.** | | | |
| --- | --- | --- | --- |
| **Characteristic, mean ± SD** | **Baseline HAART (N=173)** | **No baseline HAART (N=172)** | **P-value** |
| Total Cholesterol, mg/dL | 180.4 ± 53.0 | 157.6 ± 49.1 | <0.001 |
| Triglycerides, mg/dL | 118.8 ± 60.2 | 120.2 ± 56.8 | 0.42 |
| LDL-C, mg/dL | 97.6 ± 41.6 | 88.6 ± 33.6 | 0.03 |
| HDL-C, mg/dL | 48.8 ± 16.3 | 41.3 ± 17.9 | <0.001 |
| Waist Circumference, cm | 74.8 ± 15.8 | 73.4 ± 10.6 | 0.17 |
| BMI, kg/m^2^ | 22.9 ± 5.4 | 21.21 ± 4.1 | <0.001 |
| SBP, mmHg | 113.5 ± 16.6 | 111.8 ± 18.8 | 0.18 |
| DBP, mmHg | 72.8 ± 13.0 | 72.4 ± 13.4 | 0.37 |

LDL-C= Low Density Lipoprotein Cholesterol HDL-C= High Density Lipoprotein Cholesterol BMI= Body Mass Index SBP= Systolic Blood Pressure DBP= Diastolic Blood Pressure

SSTable 2: Basic characteristics of study population.

|  | **HIV treatment status** | |  |  |
| --- | --- | --- | --- | --- |
| **Characteristic** | **Baseline HAART** | **No Baseline HAART** | **HIV -** |  |
|  | **N=173** | **N=172** | **N= 161** | **P-value** |
| Age (years) mean ± SD | 41.7 ± 9.6 | 40.9 ± 10.6 | 32.9 ±1 0.5 | <0.001 |
| *Older Age, n (%) | 13 (8) | 23 (13.4) | 15 (9.3) |  |
| Female n (%) | 136 (78.6) | 113 (65.7) | 45 (28.0) | <0.001 |
| Christianity, n (%) | 159 (91.9) | 153 (89.0) | 141 (87.6) | 0.415 |
| Employed, n (%) | 146 (84.4) | 146 (84.9) | 126 (78.3) | 0.21 |
| BMI, mean± SD | 22.9 ± 5.5 | 21.2 ± 4.1 | 26.2 ± 4.4 |  |
| Underweight, n (%) | 29 (16.8) | 44 (25.6) | 2 (1.2) | <0.001 |
| Normal, n (%) | 98 (56.7) | 103 (59.9) | 69 (42.9) |  |
| Overweight, n (%) | 29 (16.8) | 17 (9.9) | 65 (40.3) |  |
| Obese, n (%) | 17 (9.8) | 8 (4.7) | 25 (15.5) |  |
| Hemoglobin (g/dL),mean± SD | 11.2 ± 1.9 | 10.3 ± 2.1 | 13 ± 1.4 | 0.001 |
| CD4.count,(IQR) cells/μL | 323 (120,536) | 164 (56,290) | NA | <0.001 |
| HIV type, n (%) I | 163 (94.2) | 163 (94.8) | NA | 0.497 |
| II | 6 (3.5) | 3 (1.7) | NA |  |
| I & II | 4 (2.3) | 6 (3.5) | NA |  |
| WHO HIV Staging n, %, I | 22 (12.7) | 14 (8.1) | NA | 0.04 |
| II | 37 (21.4) | 21 (12.2) | NA |  |
| III | 101 (58.4) | 122 (70.9) | NA |  |
| IV | 13 (7.5) | 15 (8.7) | NA |  |
| EFV based regimen n, % | 96 (54.2) | NA | NA | NA |
| NVP based regimen n, % | 86 (49.7) | NA | NA | NA |

*Older Age=male >45 years, female>55 years, …means not applicable, EFV = Efavirenz, NVP=Nevirapine

|  |  | | **Unadjusted** | | **Adjusted** | | |  |
| --- | --- | --- | --- | --- | --- | --- | --- | --- |
| **CVD risk factor** | **Individuals with HIV**  **On HAART**  **with**  **CD4 count >350** | **Blood donors without HIV** | | **OR (95%CI)** | | **P-value** | **OR (95%CI)** | **P-value** |
|  | **N =77** | **N = 161** | |  |  |  |  |  |
| Hypertension, n (%) | 7 (9.0) | 8 (5.0) | | 1.9 (0.7 - 5.5) | | 0.23 | 1.1 (0.3-4.5) | 0.89 |
| Hypercholesterolemia, n (%) | 27 (40.3) | 24 (15.4) | | 3.7 (1.9 - 7.1) | | <0.001 | 2.2 (0.9 - 5.2) | 0.09 |
| Diabetes mellitus, n (%) | 3 (4.5) | 1 (0.6) | | 7.5 (0.8 -73) | | 0.08 | 2.8 (0.2 - 40.7) | 0.44 |
| Hypertriglyceridemia, n (%) | 20 (34.5) | 31 (21.1) | | 2.0 (1.0 - 3.8) | | 0.05 | 2.3 (0.9 - 6.2) | 0.09 |
| High LDL-C, n (%) | 5 (8.8) | 5 (3.4) | | 2.7 (0.8 - 9.9) | | 0.12 | 3.6 (0.6 - 21.5) | 0.16 |
| Low HDL-C, n (%) | 25 (40.3) | 99 (66.9) | | 0.3 (0.2 - 0.6) | | <0.001 | 0.3 (0.1 - 0.8) | 0.01 |
| Smoking, n (%) | 2 (2.6) | 2 (1.2) | | 2.1 (0.3 - 15.3) | | 0.46 | 5.1 (0.4 - 65.6) | 0.20 |
| Abdominal obesity, n (%) | 30 (39.0) | 31 (19.2) | | 2.7 (1.5 - 4.9) | | 0.001 | 1.3 (0.5 - 3.6) | 0.65 |
| Any abnormal risk factor | 67 (87.0) | 128 (79.5) | | 1.7 (0.8 – 3.7) | | 0.16 | 1.1 (0.4 – 3.1) | 0.84 |
| >1 CVD risks | 53 (68.8) | 62 (38.5) | | 3.5 (2.0 – 6.3) | | <0.001 | 2.1 ( 0.9 – 4.6) | 0.08 |
| >3 CVD risks | 5 (6.5) | 3 (2) | | 3.7 (0.9- 15.8) | | 0.08 | 4.6 ( 0.7 – 29.8) | 0.11 |

**SSTable 3**: Cardiovascular risk factors among HIV individuals on HAART with CD4 count>350 and blood donors without HIV

Adjusted parameters=adjusted for age, sex, and body mass index. LDL-C= Low-density lipoprotein cholesterol, HDL-C= High-density lipoprotein cholesterol, CVD = Cardiovascular disease, OR= odds ratio. Hypercholesterolemia was defined as total cholesterol ≥ 200 mg/dL (≥ 5.18 mmol/L) of self-reported use of lipid lowering therapy. Hypertriglyceridemia was defined as triglycerides ≥ 150 mg/dL (≥ 1.7 mmol/L). Low HDL cholesterol was defined as HDL-C ≤ 50mg/dL (≤ 1.30 mmol/L) for women or ≤ 40 mg/dL (≤1.04 mmol/l) for men. High LDL cholesterol was defined as LDL-C ≥ 150mg/dL (≥3.8mmol/L). Abdominal obesity was defined as a waist circumference of >80 cm in females and >94 cm in males.

**SSTable 4**: Cardiovascular risk factors of HIV individuals with CD4 count <350 and HIV individuals with CD4 count >=350 compared to blood donors without HIV

| **CVD risk factor** | **Unadjusted** | | | | **Adjusted** | | | |
| --- | --- | --- | --- | --- | --- | --- | --- | --- |
|  | **HIV with CD4<350** | | **HIV with CD4>=350** | | **HIV with CD4<350** | | **HIV with CD4>=350** | |
|  | **OR(95%CI), relative to Blood donors** | p-value | **OR(95%CI), relative to Blood donors** | p-value | **OR(95%CI), relative to Blood donors** | p-value | **OR(95%CI), relative to Blood donors** | p-value |
| Hypertension | 1.4(0.6-3.3) | 0.44 | 3.0(1.2-7.5) | 0.02 | 0.9(0.3-2.4) | 0.78 | 1.3(0.4-3.9) | 0.62 |
| Hypercholesterolemia | 1.8(1-3.1) | 0.03 | 3.4(1.8-6.4) | <0.001 | 1.3(0.7-2.7) | 0.38 | 2.1(1.0-4.4) | 0.05 |
| Diabetes mellitus | 5.7(0.7-45.8) | 0.10 | 14.1(1.7-116.5) | 0.01 | 6.4(0.7-58.5) | 0.10 | 14.8(1.5-142.5) | 0.02 |
| Hypertriglyceridemia | 1.1(0.7-1.9) | 0.62 | 1.9(1-3.5) | 0.05 | 1.1(0.6-2.2) | 0.75 | 1.6(0.7-3.5) | 0.23 |
| High LDL-C | 1.9(0.6-5.5) | 0.24 | 3.6(1.1-11.5) | 0.03 | 1.6(0.5-5.7) | 0.44 | 2.7(0.7-10.5) | 0.15 |
| Low HDL-C | 0.6(0.4-1.0) | 0.06 | 0.4(0.2-.07) | 0.001 | 0.5(0.3-0.9) | 0.02 | 0.3(0.2-0.7) | 0.002 |
| Smoking | 1(0.2-5.9) | 0.98 | 2.6(0.4-15.6) | 0.31 | 2.2(0.3-17.2) | 0.46 | 6.7(0.8-55.9) | 0.08 |
| Abdominal obesity | 0.6(0.3-1.0) | 0.06 | 2.5(1.4-4.4) | 0.001 | 0.3(0.1-0.7) | 0.007 | 1.0(0.4-2.5) | 0.93 |
| Any abnormal risk factor | 1.4(0.9-2.5) | 0.14 | 1.8(0.9-3.7) | 0.10 | 1.0(0.5-1.9) | 0.95 | 1.1(0.5-2.6) | 0.78 |
| >1 CVD risks | 1.7(1.1-2.5) | 0.01 | 3.5(2.0-6.0) | <0.001 | 1.3(0.8-2.3) | 0.30 | 2.3(1.2-4.3) | 0.01 |
| >3 CVD risks | 1.5(0.4-6.00 | 0.55 | 4.8(1.2-18.5) | 0.02 | 1.8(0.4-8.20) | 0.44 | 4.5(1.0-20.3) | 0.05 |

Adjusted parameters=adjusted for age, sex, and body mass index. LDL-C= Low-density lipoprotein cholesterol, HDL-C= High-density lipoprotein cholesterol, CVD = Cardiovascular disease, OR= odds ratio. Hypercholesterolemia was defined as total cholesterol ≥ 200 mg/dL (≥ 5.18 mmol/L) of self-reported use of lipid lowering therapy. Hypertriglyceridemia was defined as triglycerides ≥ 150 mg/dL (≥ 1.7 mmol/L). Low HDL cholesterol was defined as HDL-C ≤ 50mg/dL (≤ 1.30 mmol/L) for women or ≤ 40 mg/dL (≤1.04 mmol/l) for men. High LDL cholesterol was defined as LDL-C ≥ 150mg/dL (≥3.8mmol/L). Abdominal obesity was defined as a waist circumference of >80 cm in females and >94 cm in males.
